# Comparison of the Rheological Properties of Plant Proteins from Various Sources for Extrusion Applications

**DOI:** 10.3390/foods10081700

**Published:** 2021-07-22

**Authors:** Patrick Wittek, Goeran Walther, Heike P. Karbstein, M. Azad Emin

**Affiliations:** 1 Institute of Process Engineering in Life Sciences, Chair of Food Process Engineering, Karlsruhe Institute of Technology, 76131 Karlsruhe, Germany; patrick.wittek@kit.edu (P.W.); heike.karbstein@kit.edu (H.P.K.); 2 General Mills, R&D, James Ford Bell Technical Center, Golden Valley, MN 55427, USA; goeran.walther@genmills.com

**Keywords:** plant protein, extrusion, rheological properties, viscoelasticity, closed cavity rheometer

## Abstract

Plant proteins in foods are becoming increasingly popular with consumers. However, their application in extruded products remains a major challenge, as the various protein-rich raw materials (e.g., from different plant origins) exhibit very different material properties. In particular, the rheological properties of these raw materials have a distinct influence on the extrusion process and must be known in order to be able to control the process and adjust the product properties. In this study, process-relevant rheological properties of 11 plant-based protein-rich raw materials (differing in plant origin, protein content, and manufacturer) are determined and compared. The results demonstrate distinct differences in the rheological properties, even when plant origin and protein content are identical. Time sweeps reveal not only large differences in development of viscosity over time, but also in magnitude of viscosity (up to 15-fold difference). All materials exhibit gel behaviour and strain thinning behaviour in the strain sweeps, whereas their behaviour in the non-linear viscoelastic range differs greatly. Typical relaxation behaviour of viscoelastic materials could be observed in the stress relaxation tests for all materials. Comparison of the maximum achieved shear stress, which correlates with the elastic properties, reveals an up to 53-fold difference. The results of this study could serve as a starting point for adapting raw material selection and composition to process and product design requirements and help to meet the challenge of applying plant-based proteins in food extrusion.

## 1. Introduction

Interest in the use of plant proteins in extruded foods is steadily increasing [1,2]. Protein-rich raw materials from plant origin can be used either as the main ingredient, as in some meat analogues [3,4,5,6], or to increase the protein content of traditional starch-based products, such as in breakfast cereals or snacks [7,8,9].

These protein-rich raw materials, i.e., flours, concentrates, and isolates, can be produced from various plants. The most commonly used raw materials in food extrusion are from soybean, wheat, and pea [10]. However, recent developments have also demonstrated the potential use of many other plant protein sources for extrusion applications, such as oat [11], canola [12], lupin [13], peanut [14,15,16], and hemp [17].

The proteins in these raw materials differ in their amino acid composition and molecular structure [18,19]. Both parameters are initially determined by the plant origin of the raw material, e.g., the plant species or the growth conditions of the plant. However, the molecular structure of the proteins can be strongly influenced by further processing steps necessary to produce the protein-rich raw materials, such as the removal of lipid and polysaccharide components [20]. Thus, a variety of resulting molecular structures and significant differences in the material properties of the raw materials arises [19,21], even if the plant origin and protein content are the same [5,22,23,24].

In particular, the rheological properties of these raw materials have a distinct influence on the extrusion process. For example, the shear stresses generated by screw rotation, which are responsible for physical and chemical changes, are a function of viscosity [25,26]. In processes where product structure is determined by expansion at the die exit (such as directly expanded cereals), structuring depends on elasticity in addition to viscosity [27,28,29,30]. And the flow behaviour in the cooling die, which is important for the product structure of high moisture extruded meat analogues, is also a function of the rheological properties [3,31,32,33].

In order to control the process and adjust the product properties, it is therefore necessary to know how the rheological properties are determined by the raw material selection. However, rheological measurements at process-relevant conditions, i.e., at high temperatures (>100 °C) and high protein concentrations, pose a challenge. A closed cavity rheometer has been proposed for this task [34,35,36] as it overcomes many of the limitations of conventional methods for this purpose. This device has already been applied to measure the rheological properties of diverse plant-based protein-rich raw materials [3,34,36,37,38,39,40,41,42,43,44,45]. These studies were limited to conventional protein sources, such as soybean, wheat, and pea. Furthermore, the measurement conditions (such as temperature, water content, etc.) are very different, so that a comparison between the different study results is only possible to a limited extent. Thus, it is not known how strongly the rheological properties of different raw materials can vary and therefore it is not possible to estimate how strongly the process and product are influenced by the raw material selection or composition.

The objective of this work is therefore to determine and compare the rheological properties of various plant-based protein-rich raw materials. In order to cover as broad a spectrum as possible, 11 different, commercially available raw materials were selected for this work. Five of them are from soy, and the other six from pea, oat, rice, potato, wheat, and canola. The selection of soy raw materials should cover different manufacturing processes, and the six other raw materials should cover different plant origins. The rheological properties of the raw materials are determined with a closed cavity rheometer at a moisture content of 30% (*w*/*w*) and a temperature of 120 °C, which corresponds to typical extrusion conditions. First, the development of viscosity over time will be observed. Subsequently, the strain-dependent rheological behaviour will be determined and compared by means of strain sweeps and the (visco-)elastic properties by means of stress relaxation tests.

## 2. Material and Methods

### 2.1. Material

The investigated plant-based raw materials are listed in Table 1. Protein contents are given by the manufacturers. Moisture content was determined gravimetrically by weighing a defined amount of material before and after drying at 105 °C until mass-consistency. Short names given in the first column will be used hereafter.

### 2.2. Dough Preparation

Doughs for the rheological measurements were prepared by mixing the material with deionized water (Millipore Sigma, Burlington, USA) in a Thermomix (Vorwerk, Wuppertal, Germany) to achieve a moisture content of 30% (*w*/*w*), taking into account moisture content of raw material. Doughs were vacuum-sealed and stored in a refrigerator at 8 °C for at least 16 h to ensure equilibrium of hydration. All doughs were prepared in duplicate and all measurements were performed at least three times per mixture.

### 2.3. Rheological Measurements

**Figure 1:**
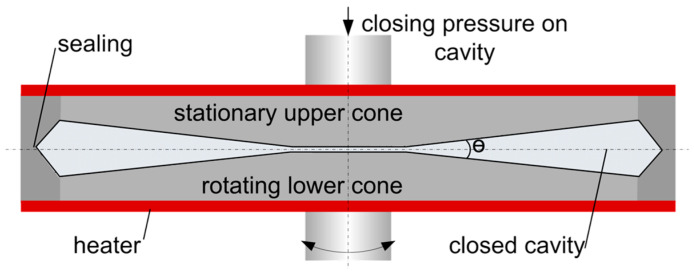
Closed cavity rheometer used for rheological measurements (picture taken from Emin & Schuchmann, 2017 [35]).

Rheological measurements were performed using a closed cavity rheometer (CCR) from TA Instruments, Inc. (New Castle, DE, USA), as shown in Figure 1. Since details on the device are described elsewhere [34,35,45], only a brief description is given here. The dough (approximately 5.5 g per measurement) is placed between the two cones and the cavity is closed by moving the cones together. The cavity is pressurized up to 4500 kPa, which prevents moisture loss and allows measurement at high temperatures. The resulting force from the sinusoidal rotational deformation of the lower cone is monitored and used for calculation of the rheological properties.

#### 2.3.1. Time Sweeps

In isothermal time sweep analyses, the material is treated at a defined temperature and the corresponding rheological properties are measured as a function of time. This methodology has already been used, for example, to track polymerization and degradation reactions in highly-concentrated wheat gluten [45]. In the present study, time sweep measurements were performed at 120 °C for 120 s, with an oscillatory shear deformation defined by a strain of 0.98% and a frequency of 1.0 Hz, which equals a shear rate of 0.06 s^−1^. According to the strain sweeps, frequency and strain of the time sweeps were in the linear viscoelastic range (LVE) for all materials, so that no effect of the measurements shear deformation on the material is ensured.

#### 2.3.2. Strain Sweeps

In the strain sweep measurements, the sample is treated at a defined temperature and frequency of oscillatory shear deformation, and the strain of deformation is step-wisely increased. This allows analysis of material behaviour in the linear viscoelastic range (at small strains), but also in the non-linear viscoelastic range (at high strains), which is expected to be especially relevant for process behaviour in extrusion [41]. In the present study, the strain sweep measurements were performed at 120 °C, with a frequency of 1.0 Hz, after a pre-treatment of the material for 60 s at 120 °C, 0.98% and 1.0 Hz. The short pre-treatment ensures that sample temperature is constant when the strain sweep starts.

#### 2.3.3. Stress Relaxation Tests

Stress relaxation tests were used to investigate elastic properties of the materials. For this purpose, the material experiences a sudden step-strain, and this step-strain is maintained by the device; the required stress is then determined as a function of time. In the present study, stress relaxation tests were performed in two steps at 120 °C. In the first step, material was pre-treated for 60 s at 0.98% and a frequency of 1.0 Hz to ensure constant temperature of the sample. In the second step, a pre-step strain of 0.1% was applied for 15 s, before the actual sudden step strain of 80% was maintained for 105 s and the required stress measured.

## 3. Results and Discussion

### 3.1. Development of Material Viscosity over Time

**Figure 2:**
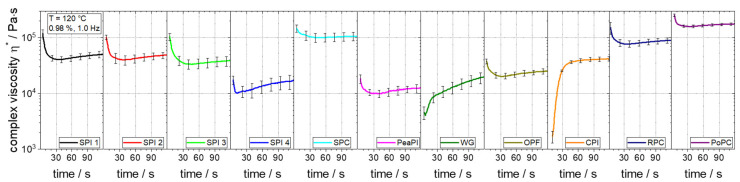
Time sweeps at 120 °C and 0.98%/1.0 Hz of different raw materials at a moisture content of 30%.

In an extrusion process, protein-rich raw materials are subjected to high thermal and mechanical stresses [46]. These can cause reactions of the present proteins, which affect the rheological properties [36,44]. Thus, the rheological properties are not only a function of temperature and shear rate, but also of the processing time. Therefore, time sweeps were performed at extrusion-like conditions (i.e., high temperature and high raw material concentration), and the development of viscosity over time was observed. Clear differences were found in these time sweeps between the raw materials (Figure 2).

Except for WG and CPI, most of the raw materials have a very similar curve shape. The viscosity decreases at the beginning, as measurement starts immediately after closing of the cavity, but the sample first heats up to the measuring temperature (i.e., from room temperature to 120 °C). Subsequently, the viscosity curve flattens out considerably and changes to a comparatively small increase in viscosity over time. The highest increase over time is observed here with SPI 4 (41 kPa·s to 50 kPa·s), while the other raw materials show lower viscosity increases.

In contrast, the curve shapes of WG and CPI are distinguished by a significant increase in viscosity. The viscosity of WG increases 5-fold from 4 kPa·s to 20 kPa·s in two minutes, while a more than 20-fold increase in less than a minute from 2 kPa·s to 42 kPa·s can be observed for CPI. Presumably, the treatment conditions cause reactions of the proteins which are accompanied by an increase in molecular weight and thus viscosity [47]. Wheat proteins, for example, are known to polymerize under similar conditions [44,45,48]. These protein reactions do not seem to take place, or only to a very small extent, in those raw materials for which only a small viscosity increase is seen. On the one hand, this could be due to the fact that the temperature is not high enough to induce reactions. On the other hand, the thermomechanical and environmental stresses (e.g., enzymatic and pH treatment) in the manufacturing process of the raw materials could have influenced the (native) protein structure and thus led to a reduction or loss of reactivity [21,49,50,51].

These differences in viscosity development would also have consequences for the development of rheological properties in the extrusion process. For less reactive materials, such as the investigated SPI or PeaPI, only local conditions such as the material temperature would be decisive for the rheological behaviour in the extrusion process. However, the rheological behaviour of reactive materials, such as the investigated WG and CPI, is additionally a function of the processing history, e.g., the process residence time.

The investigated raw materials not only show differences in the development of viscosity over time but also in the magnitude of viscosity. As a representative value, the viscosity value after a treatment time of 60 s will be compared, which corresponds to a typical average residence time in a food extrusion process [52,53]. In the comparison of soy-based protein isolates, viscosity ranges from 14 kPa·s (SPI 4) to 44 kPa·s (SPI 1), with SPI 2 (43 kPa·s) and SPI 3 (34 kPa·s) in between. These differences can probably be attributed to different manufacturing processes, since plant origin and protein content are the same [22,23]. The thermomechanical and environmental stresses in the manufacturing process can in fact not only lead to a reduction in reactivity, as mentioned, but can also be used specifically to adjust the physicochemical and thus the rheological properties of the raw materials [22]. The SPC has at least twice the viscosity (101 kPa·s) of the different SPIs. On the one hand, this could be due to differences in protein structure, as the manufacturing process has much less influence on the protein structure of SPC than in the case of SPI [49]. On the other hand, the protein content is lower (67% instead of 90%), and the proportion of polysaccharides is thus significantly higher [49,54,55,56]. The viscosity-increasing effect of polysaccharides has already been shown for a comparable system [34] and could also be the reason for the higher viscosity of SPC here.

When all raw materials are compared, the viscosity ranges from 11 kPa·s for PeaPI to 165 kPa·s of PoPC, which is a 15-fold difference. These differences could be due in part to the plant origin, which directly affects amino acid composition and molecular structure [18,19]. However, the polysaccharide component also seems to have a major influence here, as the raw materials with the highest viscosity, RPC (80 kPa·s) and PoPC, also have a comparatively high polysaccharide content. OPF (22 kPa·s) has a high polysaccharide content, but at the same time a high lipid content (17%) [11], which could be the reason for the low viscosity: the viscosity-reducing effect of lipids in comparable systems is well known [40,57].

Such large differences in viscosity as those determined (up to 15-fold) would be expected to have a significant effect on the extrusion process. For example, an increase in viscosity would lead to a proportional increase in shear stresses, significantly affecting physical and chemical changes, especially in the screw section [26]. Since the process pressures are also a direct function of the viscosity, the expansion behaviour and thus the product properties would also be largely influenced.

### 3.2. Comparison of Strain-Dependent Rheological Behaviour

**Figure 3:**
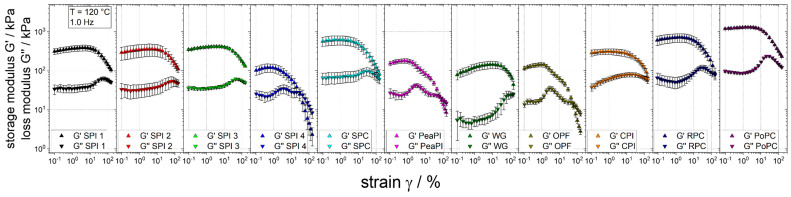
Strain sweeps at 120 °C and 1.0 Hz of different raw materials at a moisture content of 30%.

The time sweeps in this study are carried out at a constant low frequency and strain of deformation to ensure that the mechanical stress generated by the measurement had no (irreversible) influence on the material. However, since a wide distribution of shear rates from 0 up to 5000 s^−1^ [58,59] can prevail in extrusion processes, the rheological behaviour at high strains is also deemed important [41]. To reflect this, strain sweeps were used to determine and compare the strain-dependent rheological behaviour of the raw materials (Figure 3).

General information on material behaviour can be obtained from the curve shapes of G’ and G” as a function of the strain. In the linear viscoelastic range (LVE), i.e., for small strains, the storage modulus G’ is larger than the loss modulus G”, which indicates gel behaviour [60]. Additionally, plateau-like behaviour of G’ is apparent in the LVE, i.e., no or little dependence on strain. With increasing strain, the plateau of G’ changes to the nonlinear viscoelastic range (nLVE): here, G’ decreases significantly with increasing strain, a so-called strain thinning behaviour. Both, gel behaviour and strain thinning behaviour, are typical for highly-concentrated protein-rich raw materials [34,41].

In a comparison of the G” curves, the so-called “weak strain overshoot” effect is apparent for almost all raw materials: with increasing strain, G” first increases, reaches a maximum, and then drops again [61]. The exact trajectories of this effect (e.g., the shape and height of the maximum) differ between raw materials, suggesting differences in material behaviour [62]. However, a clear interpretation of this phenomenon remains challenging [63] and should not be discussed in depth in this study.

In order to allow quantitative comparison of the strain sweeps, the respective maximum strain value above which the curve drops (referred to as “range of LVE” in the following) will serve as a basis for the discussion. High values of the range of LVE indicate a more elastic network, i.e., the material is stronger and more resistant to strain (forces) [39,62]. For example, WG, which has the highest range of LVE (44.2%), is known to form a very elastic and stretchable protein network [64,65].

For the soy-based protein isolates, the range of LVE spans from 2.6% for SPI 4 to 20.1% for SPI 1-3, assuming that these differences are due to different manufacturing processes. The range of LVE for the SPC (5.2%) is significantly lower than for SPI 1-3, although it has twice the viscosity (Figure 2). The influence of the polysaccharide component is expected to lead to a comparatively lower range of LVE. In a previous work [34] it was shown that the addition of a polysaccharide leads to a lower range of LVE, and it was suggested that the formation of a dispersed polysaccharide phase could be the reason why the behaviour at high strains is influenced. In a comparison of all raw materials, the range of LVE spans from 1.9% for PeaPI and OPF to 44.2% for WG. Despite their high viscosity, RPC (7.4%) and PoPC (10.3%) show only a relatively low range of LVE, presumably also caused by the polysaccharide component.

These large differences in the range of LVE could, for example, have a distinct influence in extrusion processes for the production of meat analogues, as suggested in recent studies [39]. High range of LVE can be linked to an elastic/stable product texture and low range of LVE to a brittle/muddy product texture. This relationship may explain, for example, why the four raw materials with the highest range of LVE in this study (SPI 1, SPI 2, SPI 3, and WG) have already been applied in the high moisture extrusion of meat analogues [3,4,66,67], where a stable/cohesive or elastic texture of the product is desired [68].

### 3.3. Comparison of Elastic Behaviour

**Figure 4:**
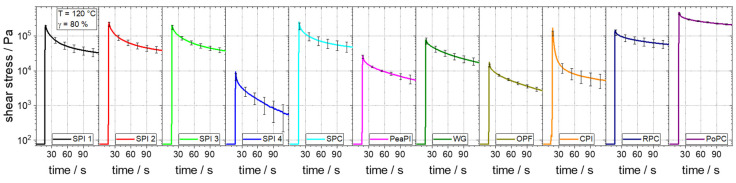
Stress relaxation tests at 120 °C and a strain of 80% of different raw materials at a moisture content of 30%.

Analogue to viscosity, elasticity also plays a major role in extrusion processes, e.g., expansion at the die outlet is significantly influenced by it [27,28,29,30]. Stress relaxation tests can be performed to investigate the (visco-)elastic behaviour of materials [69]. These tests can be a valuable enhancement in rheological characterization: they allowed, for example, to distinguish between highly concentrated materials of different origin, which was not possible with conventional oscillatory measurements [70]. Comparison of the stress relaxation test results (Figure 4) reveals clear differences in the (visco-)elastic behaviour of the raw materials.

The basic curve shape is the same for all raw materials: they exhibit a constant, relatively low value of stress up to 15 s, which is due to the constant pre-step strain of 0.1%. At 15 s, the sudden step strain leads to an immediate stress increase up to the maximum value of the curves. Thereafter, the value decreases monotonically, which is a typical behaviour for viscoelastic materials [69]. Purely elastic materials, for example, would not show a decrease in stress over time, but a constant stress.

Quantitative comparison of the stress relaxation tests will be done with the respective maximum value of the shear stress, which can be correlated with the elastic behaviour of the material [71]. For the soy-based protein isolates, this value ranges from 9 kPa for SPI 4 to 249 kPa for SPI 2, which is more than a 27-fold difference. The values of SPI 1 (202 kPa) and SPI 3 (204 kPa) rank between SPI 4 and SPI 2. These differences, which again are presumably due to the manufacturing process, are thus relatively larger than the differences in viscosities between the soy protein isolates, where there is only about a 3-fold difference (14 kPa·s to 44 kPa·s). The SPC (253 kPa) has only a slightly higher maximum shear stress than the SPI 2.

In a comparison of all raw materials, the value for the maximum shear stress ranges from 9 kPa for SPI 4 to 482 kPa for PoPC, which corresponds to a 53-fold difference and is thus also greater in relative terms than for the viscosities compared. Here, the raw materials with a comparatively low viscosity tend to have a comparatively low maximum shear stress (ergo elasticity), and raw materials with a high viscosity also tend to have a higher maximum shear stress. However, the actual order of the raw materials in the comparisons does not match. For example, CPI has a lower viscosity than RPC (39 kPa·s to 80 kPa·s), but a higher maximum shear stress (170 kPa to 144 kPa).

The differences in elasticity between the raw materials are also expected to have an impact on the extrusion process. For instance, a comparably much smaller decrease of elastic properties as the measured ones has already lead to a significant decrease of the sectional expansion in a starch-based matrix [28].

## 4. Conclusions

The objective of this work was to determine and compare the rheological properties of eleven plant-based protein-rich raw materials. The raw materials were rheologically characterized by means of time sweeps, strain sweeps, and stress relaxation tests. In the time sweeps, two raw materials (WG and CPI) showed a significant increase (up to 20-fold) in viscosity over time, while the nine other raw materials showed only a comparatively small increase in viscosity. Furthermore, the raw materials differed significantly in the magnitude of viscosity after one minute. Among the four soy-based protein isolates, viscosity ranged from 14 kPa·s to 44 kPa·s, corresponding to a 3-fold difference. Meanwhile, comparison of all raw materials revealed a 15-fold difference in viscosity (range from 11 kPa·s to 165 kPa·s). The strain sweeps showed that all the raw materials exhibited gel behaviour and strain thinning behaviour, while the quantitative dimensions of the weak strain overshoot effect varied. The values for the range of LVE for the four soy-based protein isolates range from 2.6% to 20.1%; when all raw materials are compared, this value shows a range from 1.9% to 44.2%. The stress relaxation tests could show that all raw materials exhibit the typical relaxation behaviour of viscoelastic materials. The maximum shear stress, which can be correlated with the elastic properties, ranges for the soy-based protein isolates from 9 kPa to 249 kPa, which corresponds to a more than 27-fold difference. The difference is even greater when all raw materials are compared: a 53-fold differences is apparent here (9 kPa to 482 kPa). The measurements allowed to identify distinct differences between the rheological properties of the raw materials, even when plant origin and protein content were identical. The combination of all three methods for any given raw material offers an extensive and valuable insight into rheological behaviour. Since rheological properties can have a distinct impact on the extrusion process, the presented rheological approach and the obtained results can be used to adapt the raw material selection and/or composition to process and product design requirements and help to meet the challenge of applying plant-based proteins in food extrusion.

## Figures and Tables

**Table 1:** List of investigated plant-based raw materials.

Short Name	Plant Origin	Brand Name	Manufacturer	Protein Content (Dry Basis)	Moisture Content
SPI 1	Soy	Supro ST	Solae	>90%	3.4%
SPI 2	Soy	PRO-FAM 974	ADM	>90%	2.7%
SPI 3	Soy	SUPRO 500E IP	Solae	>90%	4.8%
SPI 4	Soy	PRO-FAM 781	ADM	>90%	5.6%
SPC	Soy	Alpha 8 IP	Solae	>67%	5.1%
PeaPI	Pea	Nutralys S85 Plus N	Roquette	>84%	2.5%
OPF	Oat	PrOatein	Tate&Lyle	>50%	3.0%
RPC	Rice	Remypro N80+	Beneo	>79%	4.6%
PoPC	Potato	Protastar	Avebe	>76.5%	6.4%
WG	Wheat	Vital Wheat Gluten	Kröner-Stärke	>83%	4.5%
CPI	Canola	Canola Pro	DSM	>90%	4.6%

## Data Availability

Not applicable.
